# Serial Patterns of Ovarian Cancer Biomarkers in a Prediagnosis Longitudinal Dataset

**DOI:** 10.1155/2015/681416

**Published:** 2015-12-24

**Authors:** Oleg Blyuss, Alex Gentry-Maharaj, Evangelia-Orania Fourkala, Andy Ryan, Alexey Zaikin, Usha Menon, Ian Jacobs, John F. Timms

**Affiliations:** ^1^Women's Cancer, Institute for Women's Health, University College London, Gower Street, London WC1E 6BT, UK; ^2^University of New South Wales, Sydney, NSW 2052, Australia

## Abstract

Early detection of ovarian cancer through screening may have impact on mortality from the disease. Approaches based on CA125 cut-off have not been effective. Longitudinal algorithms such as the Risk of Ovarian Cancer Algorithm (ROCA) to interpret CA125 have been shown to have higher sensitivity and specificity than a single cut-off. The aim of this study was to investigate whether other ovarian cancer-related biomarkers, Human Epididymis 4 (HE4), glycodelin, mesothelin, matrix metalloproteinase 7 (MMP7), and cytokeratin 19 fragment (CYFRA 21-1), could improve the performance of CA125 in detecting ovarian cancer earlier. Serum samples (single and serial) predating diagnosis from 47 women taking part in the UK Collaborative Trial of Ovarian Cancer Screening (UKCTOCS) who went on to develop primary invasive ovarian, fallopian tube, or peritoneal cancer (index cancer) (170 samples) and 179 matched controls (893 samples) were included in the study. A multiplex immunobased assay platform (Becton Dickinson) allowing simultaneous measurement of the six serum markers was used. The area under the ROC curve for the panel of three biomarkers (CA125, HE4, and glycodelin) was higher than for CA125 alone for all analysed time groups, indicating that these markers can improve on sensitivity of CA125 alone for ovarian cancer detection.

## 1. Translational Relevance

Despite progress in ovarian cancer treatment over the last decade, most cases are detected at a late stage when 5-year survival is about 46.2%. Early detection is crucial to reducing mortality from the disease. Although serum CA125, which is currently the only marker used in clinical practice, has an encouraging sensitivity for detection of ovarian cancer, its level is low in early stage disease and becomes elevated only closer to the late stage. Therefore, there is a need to identify additional markers/multimarker algorithms that would improve earlier detection. Previous studies reported controversial results whether inclusion of HE4 analysis could improve the early prognosis of ovarian cancer [1, 2], and some papers also considered approaches to assessing the risk of epithelial ovarian cancer in women with a pelvic mass [3, 4]. In this study we demonstrate improved performance of models which combine CA125 with HE4 and glycodelin in earlier detection and also provide lead time. These findings will need to be validated in further independent sets but may be of value in further work trying to improve screening algorithms.

## 2. Introduction

There are over 225,000 new cases of ovarian cancer worldwide each year with over 125,000 deaths annually from the disease [5]. Ovarian cancer has a poor prognosis in view of the advanced stage at diagnosis with over 70% of patients exhibiting spread beyond the pelvis [6] and lack of specific symptoms. Currently, ovarian cancer screening is not recommended in the general population due to lack of evidence of a mortality benefit. Strategies utilising serum cancer antigen 125 (CA125) cut-off of 35 U/mL with transvaginal sonography (TVS) as a first line test have not been shown to be effective in reducing the mortality in the Prostate Lung Colorectal and Ovarian (PLCO) Cancer screening trial [7]. Data from the multimodal arm of the UK Collaborative Trial of Ovarian Cancer Screening (UKCTOCS), which used a time-series algorithm to interpret serum CA125 (the Risk of Ovarian Cancer Algorithm (ROCA)) as a first line test followed by TVS as a second line test, has shown encouraging sensitivity and specificity of this approach on both the prevalence and incidence screening [8, 9]. While it is encouraging that 48% of the cases detected were early-stage cancers, it does raise the need for improving lead time even when high sensitivity and specificity are achieved.

Numerous other candidate serum biomarkers have been reported to improve the performance of CA125 when used in combination, although most have not been tested in longitudinal samples predating diagnosis. Possibly the best of these candidates is HE4, which has been shown to complement CA125 in discriminating ovarian cancer from benign disease and to aid in earlier detection [10–17]. In a study nested within PLCO, HE4 was the second best marker after CA125 with a sensitivity of 73% compared to 86% for CA125 [16]. Other serum markers, which include mesothelin [14, 18], matrix metalloproteinase 7 (MMP7) [11, 16, 19], cytokeratin 19 fragment (CYFRA 21-1) [16, 20], and glycodelin [11, 21], have shown encouraging performance in ovarian cancer, but mainly in clinical series. However, many of these may not be useful as ovarian cancer screening markers as they have been discovered in samples taken at clinical presentation. More recently, a design where the markers are evaluated using the PROBE design [22] has been suggested. We therefore sought to test the performance of serum CA125, HE4, mesothelin, MMP7, CYFRA 21-1, and glycodelin in a set of longitudinal case control samples sourced from UKCTOCS. Given that these samples predate cancer, they provide a unique resource for the assessment of early diagnostic markers. Markers were assessed alone and in combination for their ability to predict ovarian cancer cases prior to clinical diagnosis.

## 3. Materials and Methods

### 3.1. Study Details and Subjects

Between April 2001 and September 2005, 202,638 postmenopausal women aged 50 to 74 were randomised to UKCTOCS [23]. 50,640 women were randomised to the multimodal group where women underwent annual serum CA125 testing interpreted using the ROCA. According to the risk, women were triaged into normal, intermediate, and elevated risk, which guided further triage. All women were flagged by “cancer registries” with the follow-up used for this study as of March 2012. All women diagnosed with primary invasive ovarian, fallopian tube, and peritoneal cancer had their diagnosis confirmed by independent review of medical notes by the UKCTOCS Outcomes committee. UKCTOCS will soon report on the mortality benefit of screening using ROCA. The initial dataset included 60 cases and 180 controls. However, some data was later removed as the analysis was focused on Type 1 and Type 2 invasive cancer cases from the multimodal arm: data for 13 cases, including 11 borderline cases and 2 cases from the ultrasound arm, and 1 control, who had withdrawn from the study, were excluded. Thus, samples from 47 women who developed primary invasive epithelial ovarian, fallopian tube, or peritoneal cancer from the multimodal screening arm were included, of whom 43 were screen detected by ROCA (screen positive) and 4 were missed by screening (screen negatives). 24 of these cases had 5 serial samples each preceding diagnosis, whilst 23 had 1–3 samples each, giving a total of 170 case samples. Out of the 47 cases, 20 were early stage (FIGO stages I and II), while 27 were late stage (FIGO stages III and IV). In terms of histology, there were 31 serous cancers, 1 mucinous, 3 endometrioid, 2 clear cell, 3 carcinosarcoma, and 7 not specified cancers. Each case was matched on age at randomisation (within 5 years) and regional centre to 3 controls (179 women), who had no history of cancer or cancer diagnosis during follow-up. Each control had 4 to 5 serial samples available, giving a total of 893 control samples.

### 3.2. Serum Assays

All serum samples were assayed for CA125, glycodelin, HE4, mesothelin, MMP7 and CYFRA 21-1 using a proprietary multiplexed immunoassay based on Luminex technology which was developed and run by Becton Dickinson. Assays were run blind to the operator.

### 3.3. Statistical Analysis

Since cancer progression is known to be associated with the exponential growth of CA125 and other biomarkers, all biomarker measurements were modified via logarithmic transformation, as stated in [24], in the form of *Y* = log⁡(*Z* + 4), where *Z* is the value of a particular marker. Data was processed and explored in R 3.1.1 for Mac OS software with univariate and graphical analysis. In univariate analysis, a Mann-Whitney test was performed for each of the considered markers to test if the difference in distributions for cases and controls is significantly different. Multimarker models were generated by logistic regression using three different sets of biomarker measurements; (i) the last measurement closest to diagnosis, (ii) the last measurement within 6 months of diagnosis, and (iii) the last measurement at greater than 6 months before diagnosis. Evaluation of the performance of each model was based on Receiver Operating Characteristic (ROC) curve analysis, with determination of the significance of differences in areas under the curves using the method of DeLong et al. [25].

## 4. Results

### 4.1. Study Subjects

In total, 47 women with primary invasive epithelial ovarian, fallopian tube, or peritoneal cancer were included in the study. The stage distribution was 42.5% early stage for all cases (FIGO stages I and II) (41.9% early stage of only screen-detected cancers). The serial samples predated diagnosis of cancer up to 4.83 years, with all final samples/last measurements taken within 0.75 years of diagnosis. The overall mean age for cases across their samples was 65.46 years (range 52–77.4 years) and for controls was 63.6 years (range 50.33–78.83 years). Four cases were not detected by ROCA.

### 4.2. Univariate Analysis

**Figure 1:**
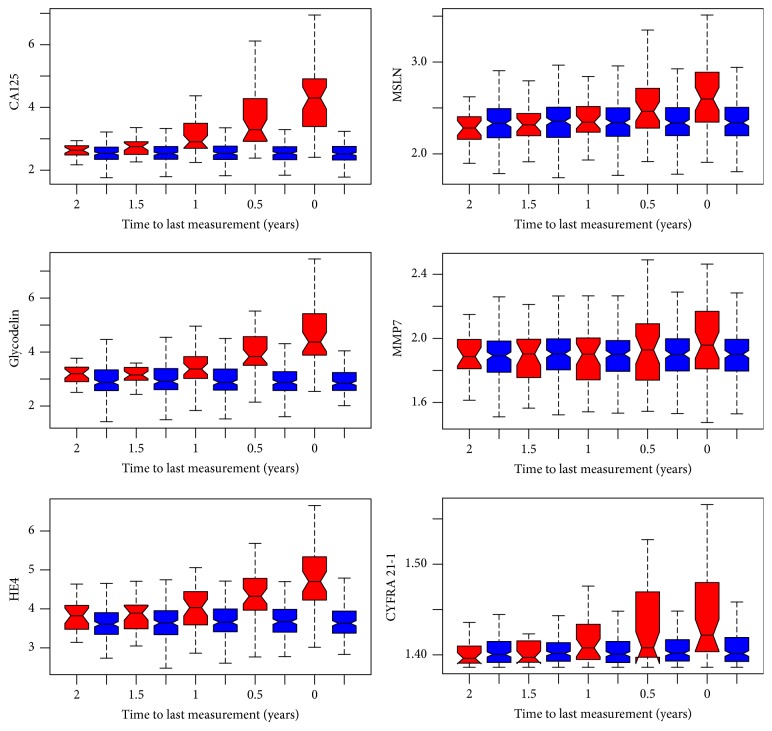
Boxplots for distributions of the values of markers in 6-month time blocks in the 2 years preceding time of last sample taken, that is, that taken closest to diagnosis. Red boxes are cases and blue boxes are controls.

**Figure 2:**
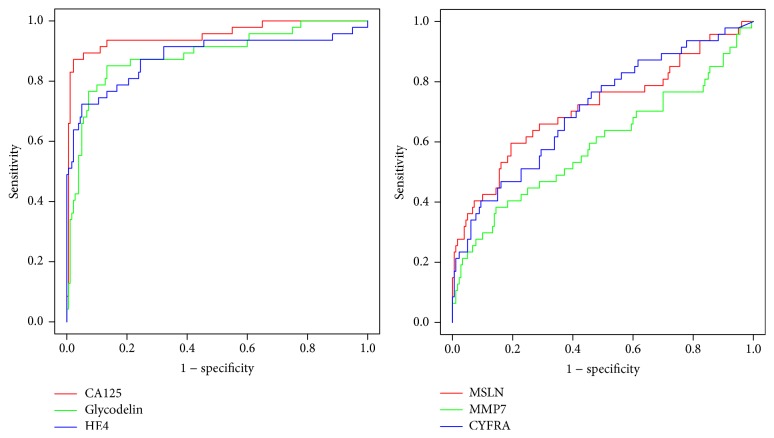
ROC curves for CA125, glycodelin, and HE4 and separately for mesothelin, MMP7, and CYFRA 21-1.

The outcome of a univariate analysis of the candidate markers is shown in Table 1. Distributions for all markers apart from MMP7 were significantly higher in cases compared to controls. Boxplots show the behaviour of the markers in 6-month steps in the 2-year period preceding the last measurement, that is, that taken closest to diagnosis, where the average time to diagnosis was 0.29 years (Figure 1). In order to obtain biomarker levels at certain time points, linear interpolation was used during the construction of boxplots. Each number on the *x*-axis in Figure 1 corresponds to both the red and blue boxplots, which represent distributions of cases and controls measurements. The figure demonstrates that CA125, glycodelin, and HE4 provide much better discrimination of cases and controls than mesothelin, MMP7, or CYFRA 21-1. Areas under the ROC curve were calculated for each marker using the last measurement for all subjects (Table 2 and Figure 2). CA125 had the highest area under the ROC curve (0.957) with glycodelin and HE4 having AUCs of >0.88.

### 4.3. Multivariate Analysis

**Figure 3:**
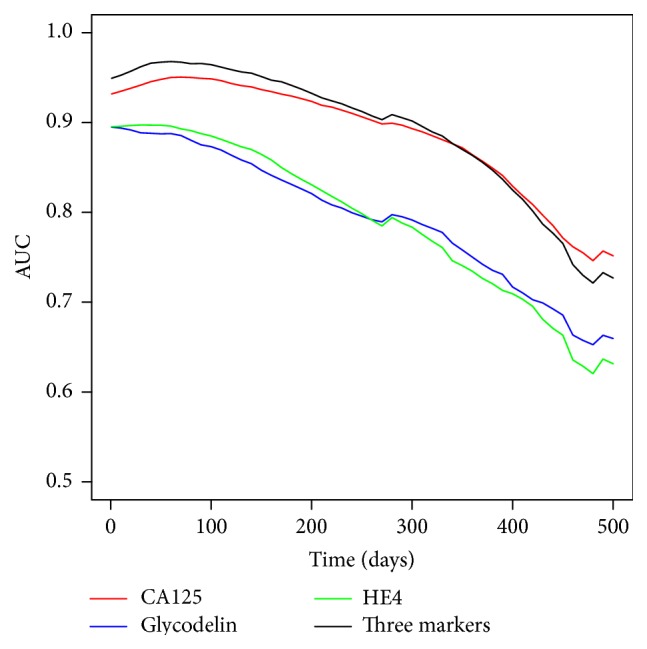
Area under the ROC curve versus time to diagnosis (linearly imputed between real measurements). The 3-marker combination (CA125, glycodelin, and HE4) consistently improves performance over CA125 alone out to 18 months prior to diagnosis.

Although CA125 had the highest sensitivity for detection of ovarian cancer compared with the other markers, we tested if combined marker models could improve on classification performance compared to CA125 alone. The results are presented in Tables 2 and 3 and Figure 3. The curves presented in Figure 3 were smoothed using linear interpolation. Classification performance of all markers and combinations increased in the lead up to diagnosis, but combinations were superior to using CA125 alone for all time groups preceding diagnosis. A model combining CA125, glycodelin, and HE4 gave an area under the ROC curve of 0.967 versus 0.957 for CA125 alone using the last measurement before diagnosis, although the increase failed significance using a threshold of *P* = 0.05 (Table 3). The AUC using samples taken >6 months from diagnosis was 0.789. The Mann-Whitney test was also performed to check if there is significant difference in distributions of screen negative cases and controls for glycodelin, HE4, and their combination. The results showed that only a difference in HE4 was significant (*P* = 0.009), while glycodelin (*P* = 0.09) and multimarker combination (*P* = 0.128) appeared not to be useful in picking up screen negatives.

## 5. Discussion

This paper reports on the performance of six ovarian cancer biomarkers, measured using a multiplex immunoassay, in a set of longitudinal prediagnosis case control serum samples sourced from UKCTOCS. We show that whilst 5 of the markers significantly discriminate ovarian cancer cases from controls at the point of last measurement prior to diagnosis, only 3 (CA125, glycodelin, and HE4) demonstrate potential for earlier diagnosis. This was reflected in the dynamics of change in time for these markers in individual cases. Combining these additional markers with CA125 improved classification performance, although the result was not significantly higher than for CA125 alone. Lack of significance of the difference could be explained by the fact that only 4 patients not detected by ROCA (in UKCTOCS) were included in this study set and future work would need to assess the performance of these markers in a more representative cohort containing ~15% ROCA-negative cases.

We show that, in addition to HE4 (which has been shown to be the second best performing marker after CA125 in the context of screening), glycodelin is a novel and useful adjunct to CA125 for early detection of ovarian cancer. Glycodelin has only been previously tested in the diagnosed case control setting [11, 21], although with promising results. This work also supports previous studies showing the potential of HE4 [10–14, 16, 19], whilst suggesting that mesothelin, MMP7, and CYFRA 21-1 are not useful markers for the early detection of ovarian cancer. The next step in this work would be to validate these findings in a larger independent set and to test the potential of these candidates in multimarker longitudinal algorithms.

## Figures and Tables

**Table 1:** Characteristics for different biomarkers from dataset. *P* values are calculated for a two-sample Mann-Whitney test.

	Cases			Controls			*P* value
	Median	Min	Max	Median	Min	Max	
CA125 (U/mL)	15	4.27	1767.53	8.7	1.54	728.29	<10^−4^
Glycodelin (ng/mL)	27.79	2.24	13356.05	13.91	<10^−4^	4763.97	<10^−4^
HE4 (pmol/L)	51.66	0.6	2378.08	33.72	<10^−4^	118.5	<10^−4^
Mesothelin (nmol/L)	7.12	1.52	44.39	6.27	0.759	17.64	0.001
MMP7 (nmol/L)	2.72	0.37	26.7	2.585	0.359	11.41	0.347
CYFRA 21-1 (ng/mL)	0.07	0	8.83	0.06	<10^−4^	2.05	0.004

**Table 2:** Areas under the ROC curve for the six individual markers with 95% confidence intervals using the last measurement for all subjects (47 case samples, 179 control samples), the last measurement for all subjects within 6 months of cancer diagnosis (42 case samples, 179 control samples), and the last measurement for all subjects at greater than 6 months before diagnosis (42 case samples, 179 control samples).

Marker	Last measurement (average 0.29 years before diagnosis)		Last measurement ≤6 months before diagnosis		Last measurement >6 months before diagnosis	
	AUC	95% CI	AUC	95% CI	AUC	95% CI
CA125	0.957	0.918–0.997	0.976	0.952–0.999	0.763	0.685–0.842
Glycodelin	0.888	0.828–0.948	0.883	0.816–0.949	0.713	0.62–0.806
HE4	0.881	0.81–0.952	0.869	0.792–0.945	0.653	0.551–0.755
Mesothelin	0.712	0.618–0.807	0.729	0.632–0.825	0.539	0.438–0.64
MMP7	0.589	0.484–0.693	0.625	0.517–0.733	0.554	0.448–0.66
CYFRA 21-1	0.71	0.622–0.797	0.72	0.634–0.805	0.492	0.406–0.61
CA125 + HE4	0.965	0.935–0.995	0.98	0.961–0.999	0.77	0.693–0.848
CA125 + glycodelin + HE4	0.967	0.938–0.996	0.982	0.966–0.998	0.789	0.714–0.865

**Table 3:** AUC and 95% confidence intervals together with sensitivity level for specificity >0.9 for CA125 alone and the best marker combination using the last measurement for all subjects. A *P* value is given for comparison of AUCs between the multimarker model and CA125 alone.

Model	AUC	95% CI	Diff. in AUCs (*P*)	Sensitivity (specificity >0.9)
CA125	0.957	0.918–0.997	—	0.894
CA125 + HE4	0.965	0.935–0.995	0.008 (0.182)	0.894
CA125 + glycodelin + HE4	0.967	0.938–0.996	0.01 (0.206)	0.915
